# Surface profile characterization of prefabricated resin composite veneers following simulated abrasion. An *in vitro* study

**DOI:** 10.4317/jced.60420

**Published:** 2023-10-01

**Authors:** Angeliki Karveli, Efstratios Papazoglou, Despina Koletsi, Maria Anagnostou

**Affiliations:** 1 Department of Operative Dentistry, School of Dentistry, National and Kapodistrian University of Athens, Greece; 2 Clinic of Orthodontics and Pediatric Dentistry, Center of Dental Medicine, University of Zurich, Zurich, Switzerland; 3 Meta-Research Innovation Center at Stanford (METRICS), Stanford University, CA, USA

## Abstract

**Background:**

The surface of resin composite veneers is susceptible to the effect of the oral environment and surface profile characterization of different veneer systems is of importance to the longevity and clinical performance of the materials. The aim of the present study was to evaluate surface profile properties, as defined by gloss and roughness parameters, of prefabricated resin composite veneers (PCV) and compare with a laboratory resin composite (LRC) system, following simulated abrasion.

**Material and Methods:**

Twenty eight composite veneers equally divided to a prefabricated composite veneer (PCV) system and a laboratory resin composite (LRC) (control group) were tested following abrasion under a toothbrush simulator. Alterations in gloss (ΔGloss) and roughness (ΔSa, ΔSz, ΔSci, ΔSdr) parameters were examined (after- before abrasion) using a glossmeter and a 3D-optical profilometer, respectively. Correlation matrices between ΔGloss and ΔRoughness parameters were sought across the two resin composite veneer groups.

**Results:**

Τhere was weak evidence that the PCV group exhibited less change in surface gloss after experimental abrasion (PCV vs LRC: mean difference ΔGloss in GU, (MD: -1.7; 95% CI: -3.3, -0.1; *p*=0.04). For the roughness parameters, ΔSci in nm3/nm2 (MD : 0.2; 95% CI: 0.1, 0.3; *p*=0.002) and ΔSdr in percentage (MD: 10.6; 95% CI: 3.7, 17.5; *p*=0.004), exhibited the most pronounced differences between the groups with strong evidence demonstrating greater changes for the PCV group compared to the LRC. No strong correlation pattern could be identified between changes in gloss and roughness parameters across the groups.

**Conclusions:**

After abrasion, both PCV and LCR showed an increase in surface gloss, while the PCV group demonstrated a rougher core surface profile than LRC.

** Key words:**Prefabricated, resin composite, veneers, gloss, roughness.

## Introduction

The integrity of resin composite restorations may be compromised as a result of chemical and mechanical interactions within the oral cavity environment (1). In the anterior region, the clinical behavior of aesthetic restorations is largely dependent on surface properties (2) and as such, finishing and polishing procedures may pose a significant long-term effect on surface quality and characterization of resin composite materials (3).

Resin composite veneers are widely used for the restoration of aesthetically compromised anterior teeth. However, they are susceptible to surface alterations as this is represented by gloss and roughness parameters (4). Prefabricated resin composite veneers have been introduced to the dental market in recent years, which, according to the manufacturers, demonstrate improved surface quality characteristics (5-8). These systems contain thin pre-polymerized composite shells and several shades of a direct composite resin for their bonding. They are customized in the mouth regarding color and shape (6). The prefabricated veneering technique is based on high pressure molding and heat curing processes, followed by laser surface vitrification. This sintering procedure enables the veneers to exhibit a hard and glossy surface (5). However, there are only few studies regarding their laboratory and clinical performance (9-11).

The purpose of this *in vitro* study was to evaluate the surface properties and surface profile characterization of a prefabricated resin composite veneer system compared to a laboratory resin composite. Specific objectives were to compare the effect of abrasion on gloss and roughness parameters between the resin composite groups, and also to test whether any correlation existed between alterations in gloss and roughness across the groups. The null hypothesis was that there is no significant difference in the effect of abrasion on gloss and roughness alterations between the groups and also, that there is no significant correlation between the two surface profile parameters tested across the groups.

## Material and Methods

The materials tested in the study were a nano-hybrid resin composite used for the fabrication of prefabricated composite veneers (PCV) (Direct Veneer, Edelweiss Dentristry, Wolfurt, Austria) and a laboratory micro-hybrid resin composite (LRC) (Sinfony, 3M ESPE, Seefeld, Germany). Glass plates (Objektträger, Waldemar Knittel Glasbearbeitungs GmbH, Braunschweig, Germany) were used to construct the specimens (a total of n=28). The surface of the glass plates was grinded with a coarse diamond (130 μm, Heico, Steinach, Switzerland). Central incisors (size: large) of PCV veneer system were bonded to half of the glass plates (n=14). More specifically, the top of the inner surface of the veneers was also grinded with a coarse diamond (130 μm, Heico). Then, the inner surface of the veneers was coated with bonding agent (Veneer Bond, Edelweiss Dentristry) and photopolymerized for 20s with a LED curing unit (1200 mW/ cm2, Elipar S10, 3M ESPE). Subsequently, a high-viscosity nano-hybrid resin composite (Edelweiss Dentristry, shade A3), which is included in the PCV kit, was used for the adaptation and bonding of the veneers on the glass plates. The resin composite was conFigured with suiTable hand instruments and the excess of the material was removed. The margins of the veneers were photopolymerized for 40s. Finishing was done with fine and extra-fine diamonds (40μm/10μm, Ηeico). Polishing was performed using sequential series of discs (Sof- Lex Finishing and Polishing extra thin Kit, 3M ESPE), one-step diamond polishers (Kenda maximus, Coltene/Whaledent AG, Altstätten, Switzerland) and polishing paste (Sparkle Diamond Polishing Paste, Pulpdent Corporation, Watertown, MA, USA). Each diamond, disc and rubber was used for the fabrication of 5 specimens and then replaced by a new one. Finally, the specimens were placed in ultrasonic bath for 3 min. The remaining 14 specimens (laboratory micro-hybrid resin composite (LRC) used as a control group) were prepared according to the instructions described below. A mold was constructed using transparent addition silicone (Star VPS Clear Bite, Danville Materials, San Ramon, CA, USA), in the size of the prefabricated veneer that was used previously. This mold was used to fabricate veneers from an indirect micro-hybrid resin composite (Sinfony, 3M ESPE) photopolymerized with a LED curing unit for 40s. Then, the veneers were bonded on the glass plates using a photopolymerizable resin cement (Variolink Esthetic LC, Ivoclar Vivadent Inc., Schaan, Liechtenstein), according to the manufacturer’s instructions. Finishing and polishing of the margins of the restorations was performed in the same way as described previously. Prior to outcome measurements, the samples were stored in distilled water, at room temperature, in dark conditions for 24 h.

For gloss measurements, a glossmeter (Novo-Curve, Rhopoint, East Sussex, UK) was used, at 60° incidence angle and a sampling area of 4.5mm. Four measurements were made for each specimen, with successive 90o rotations, and the average was calculated. A 3D optical profilometer (Wyko NT 1100, Veeco, Cambridgeshire, UK) was used to measure the surface roughness parameters of the specimens. For each sample, three consecutive measurements were performed, in VSI mode and 110 Z scan width: 100 μm, using Mirau lenses, 41.6 magnification and 2% modulation threshold.

The roughness parameters tested were: arithmetic mean deviation (Sa), maximum height of the surface (Sz), core void volume showing the volume of the surface (Sci) that could support from 5% to 80% of the bearing ratio, and the developed interfacial area ratio (Sdr).

Initial measurements of gloss and roughness were performed on PCV and LRC veneers groups bonded to glass plates. Following completion of initial measurements, all specimens were subjected to abrasion using a custom-made toothbrush simulator. For each specimen, a total of 120 min of brushing was performed using an electric toothbrush (Sonicare, Philips, Amsterdam, Netherlands), with 31000 reciprocating movements per min, twice a day with a total brushing time of 2 min (7.5’’ per tooth in dentulous patient) and vertical loading of 200 g. A standardized amount of paste, consisting of toothpaste (Colgate Total, Colgate, Manhattan, NY, USA) / water in a ratio of 1:2, was placed on the surface to be abraded. After completing the abrasion procedure, the specimens were rinsed with running water for 30s, dried for 10s and resubjected to gloss and roughness measurements. The differences between the final and initial gloss (Delta Gloss: ΔGloss) and Delta Roughness (ΔSa, ΔSz, ΔSci, ΔSdr) parameter values were calculated for the two materials tested. Correlation matrices between ΔGloss and ΔSa, ΔSz, ΔSci, ΔSdr were also sought.

Data were checked for normality of residuals for all gloss and surface roughness parameters (distributions of initial, final and differences were checked, per group) through the Shapiro-Wilk test and visually through q-q plots. Means and standard deviations were presented upon confirmation of normal distribution, while median values along with the respective interquartile range were shown in case the assumptions of normality was not met. For the assessment of differences in changes (Δ(values)) before and after abrasion between the two groups, for all surface profile characteristics, independent samples t-tests were followed (normality of distribution was confirmed in all cases). Correlation matrices between ΔGloss and Delta Roughness values (ΔSa, ΔSz, ΔSci, ΔSdr), by specimen group (PCV or LRC) were sought through the Pearson Correlation and coefficients of correlation (r) were presented. In this case, due to multiple comparisons, the level of statistical significance was adjusted with Bonferroni correction. The original level of significance was set at α= 0.05, unless otherwise reported. All statistical analyses were performed with Stata v. 15.1 (Stata Corp, College Station, TX, USA).

## Results

**Figure 1:**
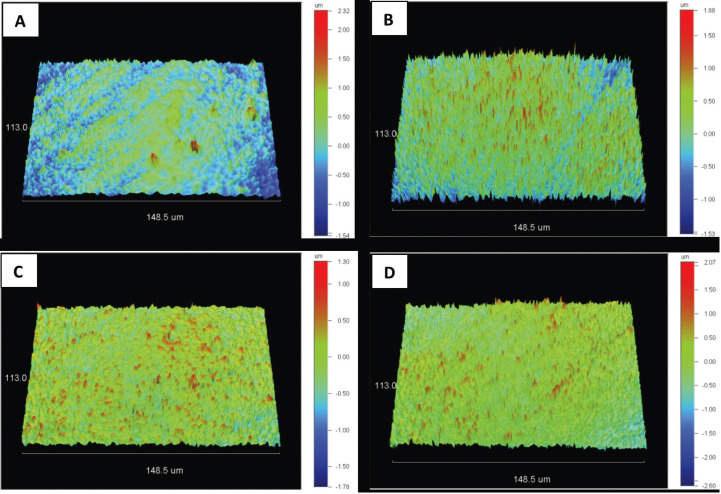
(A-D): Representative 3D optical profilometric images from tested groups. A) PCV before abrasion, B) PCV after abrasion, C) LRC before abrasion C) LRC after abrasion. Please note the differences between scale.

Descriptive statistics, either means and standard deviations, or median values and interquartile range for data distribution are presented across the groups in  Table 1. Representative 3D profilometric images are shown before and after abrasion for each group (Fig. 1A-D).

**Table 1:**
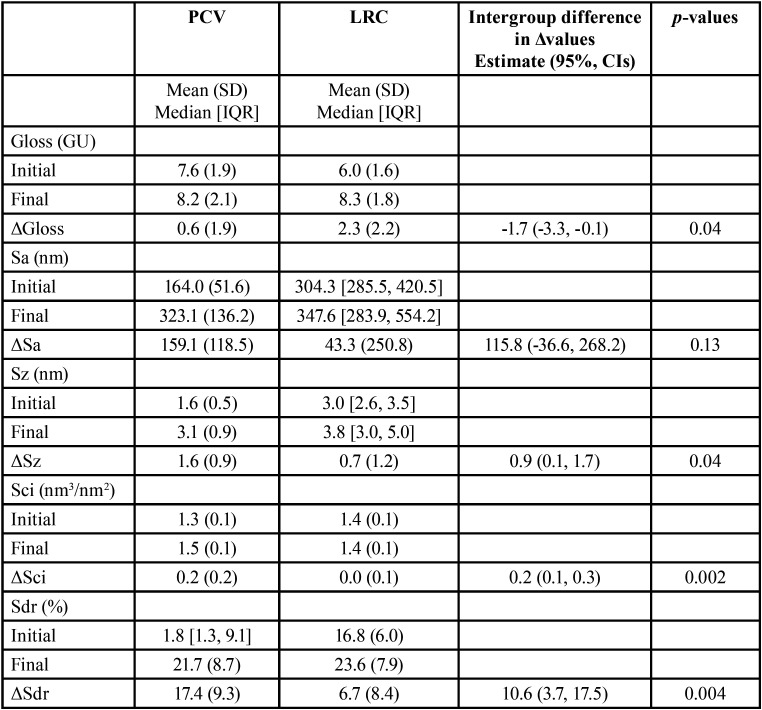
Descriptive statistics for initial, final and abrasion induced differences Δ (final- initial) values for gloss and roughness parameters per group (n=28). Inferential statistics through independent t-test intergroup comparisons are also presented for abrasion induced differences (Δfinal- initial) between the two groups, followed by presentation of the respective estimates and 95% Confidence Intervals and *p*-values.

Regarding gloss alterations (measured in Gloss Units: GU) following abrasion and comparison between the groups, there was weak evidence that the PCV group exhibited less change after experimental abrasion (PCV vs LRC: mean difference ΔGloss, MD: -1.7; 95% CI: -3.3, -0.1; *p*=0.04). For the roughness parameters, ΔSci (in nm3/nm2) and ΔSdr (%), exhibited the most pronounced differences between the groups with strong evidence demonstrating greater changes for the PCV group compared to the LRC, respectively (ΔSci: MD: 0.2; 95% CI: 0.1, 0.3; *p*=0.002 and ΔSdr: MD: 10.6; 95% CI: 3.7, 17.5; *p*=0.004). For the ΔSa and ΔSz parameters, there was either no significant difference in the alterations recorded following abrasion, or very weak evidence of differences, respectively ( Table 1).

**Figure 2:**
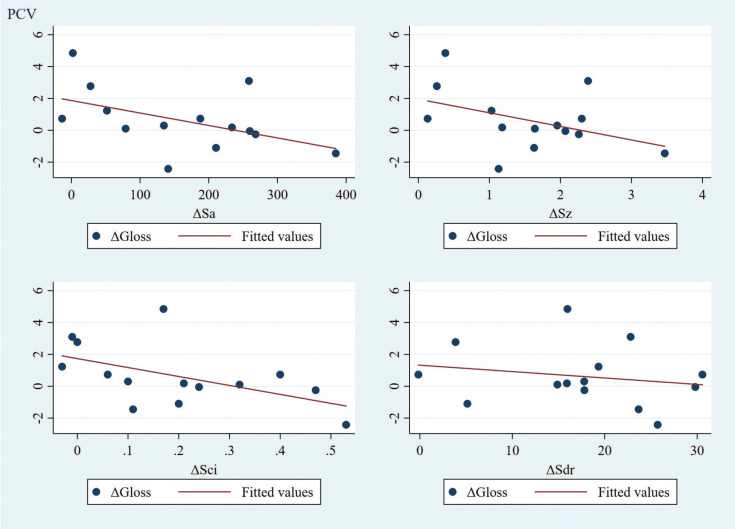
Correlation matrix for ΔGloss and ΔRoughness parameters for PCV group (n=14).

**Figure 3:**
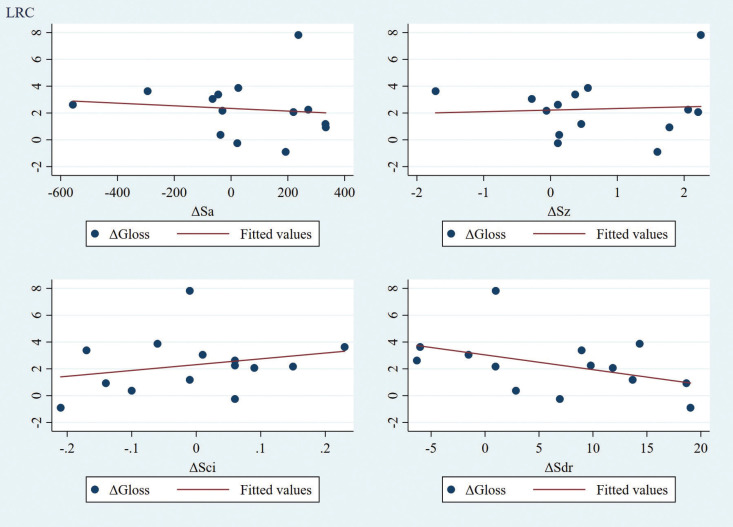
Correlation matrix for ΔGloss and ΔRoughness parameters for LRC group (n=14).

Moreover, there was no significant correlation identified between gloss and roughness parameter alterations due to abrasion for any of the groups under examination. Specifically, for the PCV group, all correlation coefficients were negative, potentially indicating a weak tendency of correlation between the gloss and roughness parameters ( Table 2, Fig. 2). No clear or consistent pattern was identified for the LRC group ( Table 2, Fig. 3).

**Table 2:**
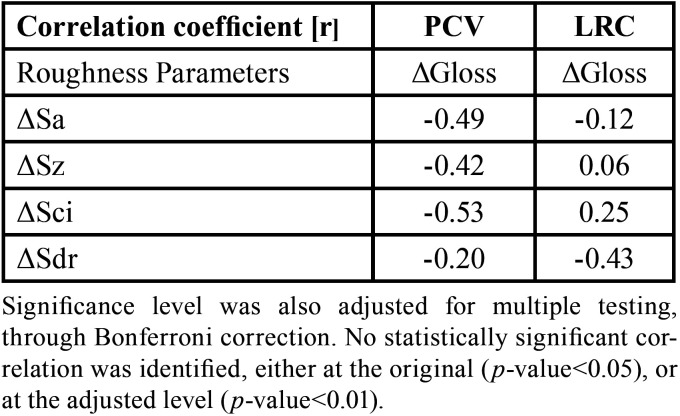
Coefficients of correlation (r) between ΔGloss and Delta Roughness values (ΔSa, ΔSz, ΔSci, ΔSdr), by specimen group (PCV or LRC).

## Discussion

Based on the findings of the present study, the null hypothesis was partially rejected, since there were differences identified between PCV and LRC groups with regard to gloss and roughness parameters, following experimental abrasion. However, no significant correlation could be documented between changes in gloss and roughness across the groups.

Both PCV and LRC groups exhibited an increase in gloss after abrasion. The flattening of the surfaces after toothbrush abrasion might be a possible explanation for this. As the veneers in both groups had a curved surface, the microscopic and macroscopic irregularities of the surface of the specimens were mostly affected by the abrasion, resulting in flatter surfaces and gloss increase. Interestingly, the increase of gloss after abrasion was greater for the micro-hybrid LRC, although we solely recorded that on the basis of weak evidence. The finding that the nano-hybrid PCV system was less affected than laboratory veneers, might be due to differences in the composition of the two materials. Although the finishing and polishing procedure was similar for both groups of materials, it is known that the abrasion of composite resins depends on their organic and inorganic components (12). The PCV system studied, consists of Bis-GMA resinous matrix and 82% w/w barium glass fillers, with particle sizes in the nano meter range (500 nm), i.e. it is a nano-hybrid composite resin (13). PCV have a special surface morphology, namely a surface coating with a thin layer of resin which is polymerized with laser. Although there is not enough evidence existing in this field of research regarding the process, it is normally implemented with high intensity and in an inert gas environment to avoid inhibition by atmospheric oxygen (5,13). The LRC system consists of a micro-hybrid composite resin, on the other hand. It contains aliphatic and cycloaliphatic monomers and two types of filler particles, 50% w/w SrAlBSiO4 glass, SiO2 and quartz of about 0.05-1 μm size (14,15). Several studies have reported that nano-filled materials may be more resistant to wear compared to micro-hybrid resin composites, being less prone to surface alterations (16,17).

In the present study, the design of the tooth abrasion method was carried out on the basis of ISO technical specifications, regarding the applied force during brushing (International Standards Organization, 1999) (18). Therefore, the maximum force used was 200g, as determined by previous studies (19,20). Other studies reported the use of a force of 100g (21), 250g, 350g (22), or 5N (23) respectively, depending on the purposes of the study. Additionally, to correspond to clinical conditions, the experimental design of the toothbrush abrasion method simulated 960 days (approximately 2.5 years) of toothbrush wear.

The RDA (Relative Dentin Abrasivity) index has been used to measure the abrasiveness of the granules contained in toothpastes, or the capacity of the toothpaste to cause abrasion in the enamel. In terms of RDA values, any value below 70 is considered to be safe for long-term use. In the present study, a common toothpaste was used, having an RDA value of 70, indicating a toothpaste with low to medium abrasive capacity (24).

During brushing, the toothpaste is diluted with saliva. Ιn the *in vitro* experiments, the toothpaste is normally diluted with distilled water, as was the case in the present study, so as to simulate, at an acceptable level, the conditions of the oral cavity. However, the unique properties of saliva, containing proteins and minerals, that would probably reduce the effect of brushing on roughness, could not be simulated (25).

Regarding alterations documented for roughness related parameters following abrasion, the nano-hybrid PCV system demonstrated more rough surface profile compared to the micro-hybrid LRC, according mostly to the functional parameter Sci and the hybrid Sdr. Notwithstanding, there was only weak evidence to support a difference in the amplitude parameter Sz in the direction of greater changes for the PCV group, or any evidence at all regarding the amplitude parameter Sa. Nano-filled resin composites are expected to present smoother surfaces compared to micro-hybrid composites after abrasion (26). However, it has been acknowledged that a number of parameters should be tested to better and more accurately characterize the surface profile of materials. The null and slight differences identified for the Sa and Sz parameters between the groups, considered simultaneously with the identified increase in these parameters after abrasion for both PCV and LRC, was indicative of a surface profile of both systems/ materials which exhibited a surface with higher peaks and/ or deeper valleys left after abrasion. The latter resulted in an increased material surface area, as also confirmed by the Sdr measurements, more pronounced in the PCV group, whatsoever. Moreover, the considerable increase in Sci solely for the PCV group indicates that the core surface structure of PCV specimens after abrasion (excluding the extreme percentage of the shallowest and deepest valleys according to the Sci definition) may contribute to an increased fluid retention capacity, thus further relating to potentially increased capacity for plaque accumulation, bacterial adhesion and biofilm formation (27). Further speculations for the identification of such intergroup differences may relate to the partial loss of the superficial glazed layer of PCV after abrasion.

A limitation of the present study is the procedure of finishing/polishing, which may have resulted in partial removal of the superficial glazed layer of PCV, thus affecting the surface profile of this material. So, the effect of finishing/polishing was probably more favorable for the micro- hybrid composite resin compared to nano-hybrid PCV.

After abrasion, the ΔSa values of the tested materials, indicating the absolute profile deviation from an average 3D surface, demonstrated no statistically significant differences, while gloss measurements, as expressed by ΔGloss showed weak evidence of significant differences between the two materials. This is consistent with the results of the study of Lai *et al*. (2018), who compared the surface properties and color stability of six flowable composite resins after abrasion. More specifically, regarding roughness, they found no significant differences between certain groups of materials, while the differences in the gloss measurements of the same materials were statistically significant and more pronounced (28). The above observations may be due to the fact that gloss, compared to roughness, is influenced by additional factors, such as the difference between refractive indices of the resinous matrix and filler particles (29). This finding may further confirm that surface gloss is a sensitive parameter in measuring the surface quality of composite resins (30).

Previous studies have described a significant correlation between gloss and surface roughness of materials (31,32). In the present study, however, there was no evidence of significant correlation regarding the alteration of gloss and roughness after abrasion, between the materials tested. The only speculation that could be made in this respect is a slight tendency for negative correlation between the two surface characterization measures, followed by the entirety of the tested roughness parameters when correlated to gloss measure. However, this was solely confirmed for the PCV group, indicating that greater roughness alterations after abrasion, characterizing the material, would be accompanied by less pronounced gloss changes.

The clinical significance of the present study resides to how the clinician handles the margins of prefabricated resin composite veneers during the finishing/polishing procedure, to minimize implications for the surface quality of the material that may bear an effect on biofilm formation and potential plaque accumulation.

Overall, after abrasion with the toothbrush simulator, both PCV and LRC showed an increase in the surface gloss. The increase was slightly greater for the LRC group. PCV presented a rougher core surface profile than LRC after abrasion. No correlation regarding gloss and roughness alteration between the tested materials could be identified.
